# Acupuncture Modulation Effect on Pain Processing Patterns in Patients With Migraine Without Aura

**DOI:** 10.3389/fnins.2021.729218

**Published:** 2021-08-26

**Authors:** Zilei Tian, Yaoguang Guo, Tao Yin, Qingqing Xiao, Guodong Ha, Jiyao Chen, Shuo Wang, Lei Lan, Fang Zeng

**Affiliations:** ^1^Acupuncture and Tuina School/The 3rd Teaching Hospital, Chengdu University of Traditional Chinese Medicine, Chengdu, China; ^2^Acupuncture and Brain Science Research Center, Chengdu University of Traditional Chinese Medicine, Chengdu, China; ^3^Hospital of Chengdu University of Traditional Chinese Medicine, Chengdu, China; ^4^Key Laboratory of Sichuan Province for Acupuncture and Chronobiology, Chengdu, China

**Keywords:** acupuncture, fMRI, resting-state, functional connectivity, migraine without aura, pain processing

## Abstract

**Introduction:**

In this retrospective study, resting-state functional connectivity (FC) in patients with migraine was analyzed to identify potential pathological pain processing patterns and compared them to those in healthy controls (HCs). The FC patterns in patients between pre- and post-acupuncture sessions were also analyzed to determine how acupuncture affects neurological activity and pain perception during the migraine interictal period.

**Methods:**

In total, 52 patients with migraine without aura (MwoA) and 60 HCs were recruited. Patients with migraine were given acupuncture treatment sessions for 4 weeks. As a primary observation, functional magnetic resonance images were obtained at the beginning and end of the sessions. HCs received no treatment and underwent one functional magnetic resonance imaging (fMRI) scan after enrollment. After the fMRI data were preprocessed, a region of interest (ROI)-to-ROI analysis was performed with predefined ROIs related to pain processing regions.

**Results:**

The first analysis showed significantly different FCs between patients with MwoA and HCs [false discovery rate corrected *p*-value (*p*-FDR) < 0.05]. The FCs were found to be mainly between the cingulate gyrus (CG) and the insular gyrus, the CG and the inferior parietal lobule (IPL), the CG and the superior frontal gyrus, and the middle frontal gyrus and the IPL. The second analysis indicated that acupuncture treatment partly restored the different FCs found in the first analysis (*p*-FDR < 0.05). Furthermore, subgroup analysis found different brain activity patterns in headache-intensity restored condition and headache-frequency restored condition. Lastly, the correlation analysis suggested a potential correlation between FCs and clinical symptoms (*p* < 0.05).

**Conclusion:**

This study suggests that pain processing is abnormal in migraine, with significantly abnormal FCs in the frontal, parietal, and limbic regions. This finding could be a typical pathological feature of migraine. Acupuncture has been identified to relieve headache symptoms in two ways: it restores the pain processing function and regulates pain perception.

## Introduction

Migraine is defined as a neurological disorder characterized by a recurrent headache that lasts 4–72 h with nausea, photophobia, and phonophobia (Headache Classification Committee of the International Headache Society (IHS), 2018). Worldwide, more than 3 billion people suffer from migraine. In fact, migraine has been identified as the sixth most prevalent of the 328 diseases and injuries evaluated in 2016 (Stovner et al., 2018). Moreover, it is among the top five Level 4 causes of years of life lived with disability in all five sociodemographic index quintiles of 1990 and 2016. When analyzed as a Level 4 cause, migraine was the second cause of disability after low back pain and ranked among the top 10 causes of a life lived with disability in all 195 countries.

Chronic pain is defined as lasting at least 3 months and not fully responding to treatment (Scholz et al., 2019). The impact on the patient and the transition of pain from acute to chronic are the challenges facing clinicians and policymakers. Pain involves changes in the spinal cord and brain at many levels (Elman and Borsook, 2016). And headache transition from episodic to chronic is the same as painful conditions affecting other parts of the body becoming chronic (Valente et al., 2021). By applying neuroimaging techniques, such as diffusion tensor imaging, and functional magnetic resonance imaging (fMRI), it is possible to identify brain functions that impact the headache experience at complex network levels (Chong et al., 2016). Functional connectivity (FC) analysis with fMRI is based on the temporal correlation of blood oxygen level-dependent (BOLD) signal fluctuations in different brain regions. These continuous low-frequency fluctuations in the BOLD signal have been considered to be functionally connected or functionally communicating (Schwedt et al., 2015). FC analysis assumes that regions are coupled or part of the same network if their functional behaviors are always interrelated. And it reflects part of the brain’s activities. Most of these brain areas include primary and secondary somatosensory cortices, the cingulate cortex, the posterior parietal cortex (PPC), and the prefrontal cortex (PFC) in migraine.

However, there is no separate migraine generator for headaches, and the brain networks interact and affect headache symptoms in a complex way (Colombo et al., 2015). Patients with migraine have different FCs of several resting-state networks compared with healthy controls (HCs), including the salience network (SN), default mode network (DMN), central-executive network (CEN), and frontoparietal attention network (FPN) (Tedeschi et al., 2013; Russo et al., 2019; Skorobogatykh et al., 2019). These networks are triggered together while performing tasks and maintain their functional organization at rest. The slow synchronized oscillations in each network are independent, very powerful, and affect the pathological mechanisms of migraine in different aspects (Shen, 2015).

As an efficacy treatment for migraine (Jiang et al., 2018; Xu et al., 2018), researchers have found that sustained acupuncture treatment could modulate functional networks, including but not limited to the DMN, FPN, limbic system, and descending pain modulatory system (Liu et al., 2021). Based on statistical inferences, these studies in functional brain networks focused on group differences between patients and HCs before and after treatment. Moreover, associations between cluster activity and clinical symptoms of headache were also noted. However, studies have rarely investigated migraine or acupuncture mechanisms from the perspective of pain processing.

This retrospective study aimed to investigate the resting-state brain activities in patients with migraine to find potential pathological pain processing patterns and compare them to those in HCs. Patterns in patients between pre- and post-acupuncture sessions were analyzed to discover how acupuncture regulates the neurological activity and affects pain perception during the migraine interictal period. To this end, this study first compared the differences in FC between patients with migraine without aura (MwoA) and HCs. Pre- and post-acupuncture treatment comparisons followed this comparison in patients with MwoA with region of interest (ROI)-to-ROI FC analysis. Furthermore, this study has also conducted a subgroup analysis by assigning patients with migraine into clinically significant improvement groups (responders) and no significant improvement groups (non-responders) to determine how acupuncture affects pain perception and migraine attacks. Finally, correlations between FCs and clinical symptoms were obtained using linear bivariate correlation.

## Materials and Methods

### Participants

In total, 52 participants with MwoA and 60 HCs were recruited from the third teaching hospital and campus of Chengdu University of Traditional Chinese Medicine.

The inclusion criteria for MwoA patients were as follows: (1) right-handedness, 17–45 years old; (2) meets the MwoA diagnosis criteria of the International Classification of Headache Disorders; (3) has migraine lasting at least 6 months, with at least one attack per month in the last 3 months; (4) has taken no migraine preventive medications for at least 3 months. Participants were excluded when one of the following criteria existed: (1) alcohol or drug abusers; (2) participant was pregnant or breastfeeding; (3) suffering from other severe psychiatric, neurological, cardiovascular, respiratory, or renal disorders; (4) suffering from other chronic pain disorders or having a history of head trauma; (5) suffering a contraindication to MRI scanning, such as claustrophobia; and (6) suffering contraindications to acupuncture, such as coagulation disorders or long-term anticoagulant medication use.

Meanwhile, HCs needed to meet the following inclusion criteria: (1) 17–45 years old; (2) right-handedness; and (3) absence of headache or other chronic pain disorders. The exclusion criteria for HCs were the same as those in patients with migraine.

All participants have undergone clinical assessment, physical examination, and laboratory tests to detect underlying diseases before recruitment. The ethics committee of the First Teaching Hospital of Chengdu University of Traditional Chinese Medicine reviewed and approved the study protocol. The study was registered on www.clinicaltrials.gov (ClinicalTrials.gov Identifier: NCT01152632). The participants provided written informed consent to participate.

### Intervention

**FIGURE 1:**
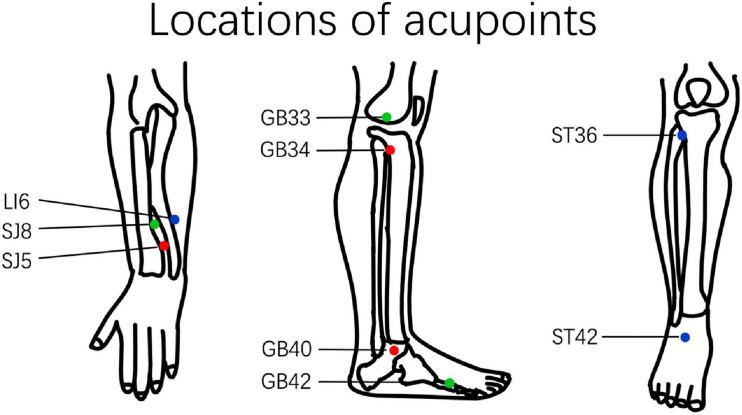
Location of acupuncture points. The different prescriptions for acupuncture points are marked using different colors. GB34, *Yanglingquan*; GB40, *Qiuxu*; SJ5, *Waiguan*; GB33, *Xiyangguan*; GB42, *Diwuhui*; SJ8, *Sanyangluo*; ST36, *Zusanli*; ST42, *Chongyang*; LI6, *Pianli*.

The intervention reported in this study followed *the standards for reporting interventions in clinical trials of acupuncture* (MacPherson et al., 2010). Only acupoints of the extremities were used to avoid the manipulation’s influence on local blood flow. Acupoint prescriptions were selected according to the participant’s Traditional Chinese Medicine diagnosis based on previous studies (Li et al., 2012). The prescription of acupoints includes the following three types: (1) *Yanglingquan* (GB34), *Qiuxu* (GB40), *Waiguan* (SJ5); (2) *Xiyangguan* (GB33), *Diwuhui* (GB42), *Sanyangluo* (SJ8); and (3) *Zusanli* (ST36), *Chongyang* (ST42), and *Pianli* (LI6) (Figure 1). All acupoints were punctured bilaterally with a 1.5 *cun* (diameter 0.25 mm, length 40 mm) filiform acupuncture needle.

Two licensed acupuncturists administered all the acupuncture treatments. The acupuncture manipulation procedure was as follows: (1) the acupuncture needles were inserted perpendicularly 5–15 mm and manipulated to achieve *deqi* sensation; (2) the needle was retained for 30 min after obtaining *deqi* sensation. The treatment sessions lasted for 4 weeks, with a total of 20 acupuncture sessions. HCs received no acupuncture treatment.

Patients agreed not to use any regular migraine medications during the study, but ibuprofen (300 mg extended-release capsules) was allowed as a rescue medication when severe pain is experienced.

### Clinical Measures

Clinical observations included duration, intensity, and frequency of headache. According to *Guidelines for controlled trials of drugs in migraine*, headache intensity and headache frequency were recorded using a headache diary (Tfelt-Hansen et al., 2012). Headache intensity was measured using a visual analog scale. Headache frequency values were obtained by recording the number of migraine attacks at least 48 h apart in 1 month after enrollment. Secondary observables included Zung Self-Rating Anxiety Scale (SAS) and the Self-Rating Depression Scale (SDS). Participants with migraine received clinical evaluations after enrollment and at the fifth week (after the 4 weeks of acupuncture sessions). HCs received clinical evaluations only during recruitment.

### MRI Scan Protocol

MRI data were collected using a 3.0T MRI scanner (Siemens 3T TrioTim, Munich, Germany) with an eight-channel phased-array head coil at the West China Hospital MRI Center of Sichuan University. The scanning schedule was as follows: HCs underwent one MRI scan after enrollment. Participants with migraine underwent MRI scans before and after the acupuncture treatment sessions (with a 4-week interval). Each scan included T1-weighted imaging and resting-state imaging.

Participants were then asked to stay awake and keep their heads still during the scan, with their eyes closed and ears plugged. The T1 images were obtained using a fast spoiled gradient recalled sequence (slice thickness = 1 mm; repetition time = 1,900 ms; echo time = 2.26 ms; field of view read = 256 mm). The BOLD images were obtained using echo-planar imaging (slice = 30; total volumes: 180; slice thickness = 5 mm; repetition time = 2,000 ms; echo time = 30 ms; field of view read = 240 mm).

### Clinical Variables Analysis

All clinical observation data from this study were statistically analyzed using SPSS 25 software (IBM Corp., Armonk, NY, United States). Between-group comparisons were then performed using independent *t*-test or Chi-square test. All statistical evaluations in this study used two-tailed tests; the significance level was α = 0.05, with a statistical threshold at *p* < 0.05 to be considered statistically significant.

According to the American Pain Society consensus statement on the clinical importance of treatment outcomes in chronic pain clinical trials, a more significant clinical symptom improvement is considered when there is a 30% improvement in pain (Dworkin et al., 2008; Fishbain et al., 2016). Thus, this study has defined the acupuncture clinically significant improvement groups (responder) as patients with a 30% improvement in headache intensity or headache frequency compared to the pretreatment evaluation.

### MRI Data Processing

#### Preprocessing Pipeline

MRI data were preprocessed with the CONN functional connectivity toolbox (20.b) (Whitfield-Gabrieli and Nieto-Castanon, 2012). The default pipeline process of the CONN toolbox includes functional sequence realignment and unwarp, slice-timing correction, outlier identification, direct segmentation and normalization, and functional smoothing (Nieto-Castanon, 2020). In the preprocessing stage, outlier identification was performed using 97th percentile normalized sampling. Then, segmentation, normalization, and resampling were performed using the default organization probability map with structural phase resolution = 1 mm, functional phase resolution = 2 mm, and half-peak full-width smoothing kernel = 4 mm.

With the CONN toolbox default denoising pipeline, noise components from the cerebral white matter, subject-motion, outlier scans or scrubbing, and constant and first-order linear session effects were regressed. Then, the band beyond 0.008–0.09 Hz was removed after regression using a band-pass filter.

#### Region of Interest Design

The ROIs selected in this study were based on the pain matrix theory (Garcia-Larrea and Peyron, 2013). Based on our previous studies (Tian et al., 2021), the second-order perceptual matrix and third-order pain memories matrix were selected as ROIs in examining the FC differences in pain and emotional processing-related regions. Due to the activity of somatosensory relevant brain regions that would bring confounding effects to the resting state, specific brain regions, including the primary and secondary somatosensory cortex, were excluded. Thus, the superior frontal gyrus (SFG), the middle frontal gyrus (MFG), the superior parietal lobule, the inferior parietal lobule (IPL), the insular gyrus (INS), the cingulate gyrus (CG), the amygdala (Amyg), the hippocampus (Hipp), and the thalamus (Tha), including the 50 subregions with a total of 100 labels, were selected as ROIs with the Brainnetome Atlas (Fan et al., 2016; Supplementary Table 1).

#### Functional Connectivity Analysis and Pearson Correlation Test

Weighted ROI-to-ROI FC analysis has characterized the condition-specific FC values among the set of ROIs mentioned above. The analysis proceeded as follows. First, connectivity matrices were computed using a weighted least-squares linear model with predefined temporal weights identifying each condition. Then, all functionally connected data were analyzed at CONN using a general linear model. Conditions and contrasts were then defined as follows:

(1) For the difference between patients with MwoA and HCs: the between-subjects specification was set to HCs + patients with MwoA (1–1), with the first scan set as the between-conditions specification.
(2) For the difference between pre- and post-acupuncture treatments: the between-subjects specification was set to patients with MwoA, with pretreatment + posttreatment (1–1) set as the between-conditions specification.
(3) For the differences between responders and non-responders in headache intensity or frequency compared with post-acupuncture, a multiple regression analysis (joint effects) was conducted; the between-subjects specification was set to patients with MwoA + acupuncture responders + non-responders ([0 1 0; 0 0 1]), with the between-conditions specification set to pretreatment + posttreatment (1–1).

Resting-state FC was considered statistically significant using standard cluster-level inferences: functional connectivity networks, with cluster thresholds defined by the multivoxel pattern analysis omnibus test, were considered statistically significant with false discovery rate corrected *p*-value (*p*-FDR) < 0.05 (Jafri et al., 2008). Subsequently, linear bivariate correlations were conducted between FC and headache intensity, headache frequency, and covariate set as gender, age, and duration of headache. The statistical threshold was considered statistically significant at a *p*-FDR < 0.05.

## Results

### Demographic and Clinical Data

In total, 52 patients with MwoA and 60 HCs finished the MRI scan. The case-control matching analysis was implemented in SPSS 25.0 to match demographic characteristics between the participants with MwoA and the HCs. After case-control matching analysis, four participants with MwoA were excluded because they could not be matched by age or gender. Thus, 48 participants with MwoA and 48 age- and sex-matched HCs were analyzed. No statistical difference was noted between the HCs and participants with MwoA in terms of gender, age, height, and weight.

Participants with MwoA had a mean headache intensity of 5.58 and a mean headache frequency of 7.00 per month at the pretreatment evaluation. At the end of the acupuncture treatment, participants with MwoA had a mean significant improvement of headache intensity of 3.76 (*p* < 0.001). Nevertheless, the mean headache frequency improvement was not significant at 6.42 per month (*p* > 0.05). Furthermore, the SAS score and SDS score were noted to significantly improve (*p* < 0.01) (Table 1).

**TABLE 1:** Demographic and clinical data of patients with migraine and healthy controls.

	**Healthy controls**	**Patients with MwoA before acupuncture**	**Patients with MwoA after acupuncture**	***p*-Value**
Female/male	37/11	37/11	–	1
Age	21.29 ± 1.89	21.17 ± 0.93	–	0.68
Height	160.92 ± 8.31	162.13 ± 6.08	–	0.42
Weight	53.80 ± 8.62	51.81 ± 7.02	–	0.22
Headache duration	–	65.83 ± 33.564	–	–
Headache intensity	–	5.58 ± 1.08	3.76 ± 1.64	0.001**
Headache frequency	–	7.00 ± 6.34	6.42 ± 5.90	0.46
SAS score	–	45.30 ± 7.97	39.734 ± 9.27	0.001**
SDS score	–	45.53 ± 10.87	41.427 ± 10.91	0.002*

Chi-square tests were applied for gender comparison, and independent-samples *t*-tests were applied to the comparison between patients with MwoA and healthy controls. MwoA, migraine without aura; SAS, Zung Self-Rating Anxiety Scale; SDS, Zung Self-Rating Depression Scale. *Independent-samples *t*-tests were significant at the 0.05 level. **Independent-samples *t*-tests were significant at the 0.001 level.

In total, 24 out of 48 participants with migraine were assigned as headache-intensity responders based on acupuncture responder definition. The demographic and clinical evaluation demonstrated no significant difference between the headache-intensity responders and non-responders before treatment (*p* > 0.05). Simultaneously, significant differences were observed between the headache-intensity responders and non-responders after treatment (*p* > 0.05) (Table 2).

**TABLE 2:** Headache intensity responder, patient with significant headache intensity improvement; non-responder, patient with nonsignificant headache intensity improvement.

	**Headache intensity responder**	**Headache intensity non-responder**	***p*-Value**
Sample size	24	24	–
Age	21.42 ± 1.89	21.33 ± 1.26	0.65
Female/male	19/5	18/6	0.74
Height	160.13 ± 7.34	163.80 ± 7.54	0.52
Weight	52.27 ± 8.37	54.54 ± 8.15	0.22
Headache duration	69.88 ± 36.25	61.79 ± 30.88	0.41
Headache intensity	5.60 ± 1.15	5.55 ± 1.03	0.85
Headache frequency	2.54 ± 1.13	4.98 ± 1.06	0.001**
Headache intensity change rate (%)	52.30	9.00	–

Independent-samples *t*-tests were applied to the comparison of the intensity of headache between the clinically significant improvement and no significant improvement groups. Headache intensity responder, intensity of headache in the clinically significant improvement group; non-responder, intensity of headache in the no significant improvement group. **Independent-samples *t*-tests were significant at the 0.001 level.

For the headache-frequency responder, 19 out of the 48 participants with migraine were assigned as headache-frequency responders. The results suggested no significant difference with regard to demographic information and headache frequency before treatment (*p* > 0.05). Nevertheless, a significant difference was found in the duration of headache between responders and non-responders (*p* < 0.01) (Table 3).

**TABLE 3:** Headache frequency responder, patient with significant headache frequency improvement; non-responder, patient with nonsignificant headache frequency improvement.

	**Headache frequency responder**	**Headache frequency non-responder**	***p*-Value**
Sample size	19	29	–
Age	21.74 ± 1.88	21.00 ± 1.87	0.19
Female/male	15/4	22/7	0.81
Height	160.79 ± 8.82	161.00 ± 8.21	0.93
Weight	52.92 ± 7.76	54.38 ± 9.23	0.57
Headache duration	50.63 ± 20.24	75.79 ± 36.99	0.01*
Headache intensity	5.63 ± 1.09	5.54 ± 1.09	0.09
Headache frequency	2.92 ± 1.43	4.31 ± 1.56	0.01*
Headache intensity change rate (%)	62.53	−68.86	–

Independent-samples *t*-tests were applied to the comparison of the frequency of headache between the clinically significant improvement and no significant improvement groups. Headache frequency responder, frequency of headache in the clinically significant improvement group; non-responder, frequency of headache in the no significant improvement group. *Independent-samples *t*-tests were significant at the 0.01 level.

### Functional Connectivity Analysis and Pearson Correlation

The quality assurance plot was checked before conducting the FC analyses. No patients with MwoA or HCs were excluded depending on the number of volumes in which the head position was 0.5 mm different from the adjacent volumes, which was more than 20% (Power et al., 2012; Supplementary Table 2).

#### The Neuroactivity Difference Between Patients With MwoA and HCs

**FIGURE 2:**
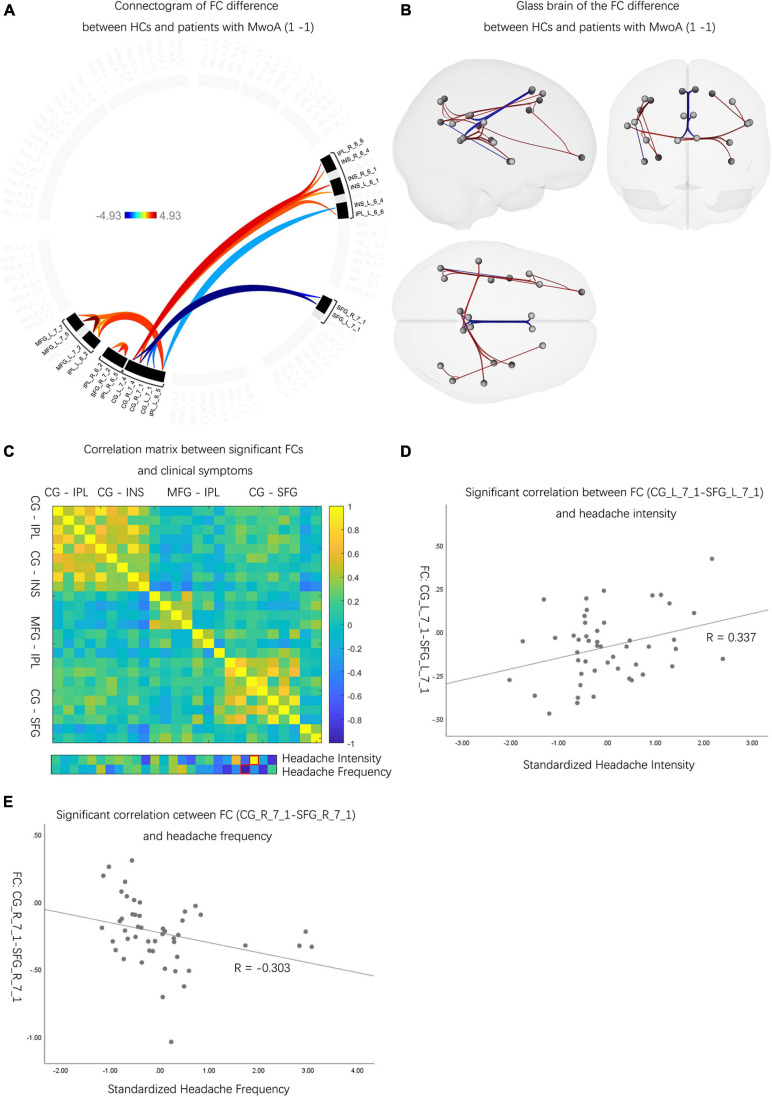
The neuro activities difference between patients with MwoA and HCs. **(A)** Patients with MwoA manifested significantly decreased and increased FCs compared with those in HCs, and the color bar represents the *t*-value of FC. **(B)** Patients with MwoA manifested significantly decreased FCs between CG and IPL, CG and INS, MFG and IPL, and within IPL subregions, MFG subregions. Moreover, patients with MwoA had significantly increased FCs between CG and SFG. Red connection lines represent an increased FC of HCs than patients with MwoA, and blue lines *vice versa*. **(C)** Two FCs were significantly correlated with headache intensity and frequency, with the red rectangle circling the significantly correlated FCs. The color bar represents the Pearson correlation coefficient, and the closer the absolute value is to 1, the higher the correlation is, and *vice versa*. **(D)** The FC between the left CG and the left SFG was significantly correlated with headache intensity (*r* = 0.337, *p* < 0.05). **(E)** The FC between the right CG and the right SFG correlated with headache frequency (*r* = –0.303, *p* < 0.05). MwoA, migraine without aura; HC, healthy control; FC, functional connectivity; L, left hemisphere; R, right hemisphere; SFG, superior frontal gyrus; MFG, middle frontal gyrus; SPL, superior parietal lobule; IPL, inferior parietal lobule; INS, insular gyrus; CG, cingulate gyrus; Amyg, amygdala; Hipp, hippocampus; Tha, thalamus.

Compared with HCs, patients with MwoA had decreased FCs between CG and IPL, CG and INS, and MFG and IPL (*p*-FDR < 0.05). Meanwhile, patients with MwoA were found to have increased FCs between CG and SFG than HCs (*p*-FDR < 0.05) (Figures 2A,B and Supplementary Table 3). Two FCs significantly correlated with headache intensity and frequency (Figure 2C). An FC between the left CG and the left SFG correlated with headache intensity (*r* = 0.337, *p* < 0.05) (Figure 2D). Another FC between the right CG and the right SFG correlated with headache frequency (*r* = –0.303, *p* < 0.05) (Figure 2E).

#### The Neuroactivity Difference Before and After Acupuncture Treatment

**FIGURE 3:**
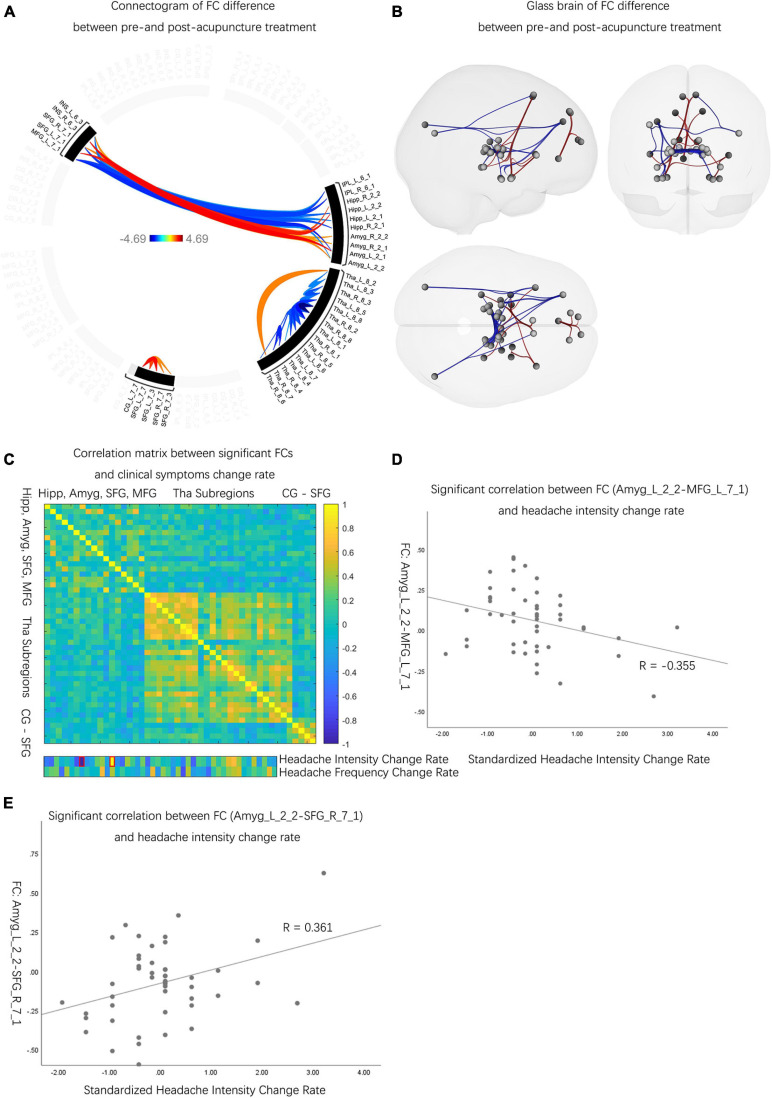
The neuro activities difference before and after acupuncture treatment. **(A)** Patients with MwoA showed significantly decreased and increased FCs between pre- and post-acupuncture, and the color bar represents the *t*-value of FC. **(B)** Compared with pre-acupuncture, patients with MwoA had decreased FCs between Amyg and INS, Amyg and SFG, CG and SFG, Hipp and SFG, and Tha subregions. Also, patients with MwoA had increased FCs between Amyg and MFG, Hipp and MFG, Hipp and INS, IPL and INS, IPL and MFG, IPL and SFG, and within Tha subregions compared with pre-acupuncture. Red connection lines represent an increased FC before acupuncture than post-acupuncture patients with MwoA, and blue lines *vice versa*. **(C)** Two FCs were significantly correlated with change rate of headache intensity, with the red rectangle circling the significantly correlated FCs. The color bar represents the Pearson correlation coefficient, and the closer the absolute value is to 1, the higher the correlation is, and *vice versa*. **(D)** The FC between the left Amyg and the left MFG was significantly correlated with headache intensity change rate (*r* = –0.355, *p* < 0.05). **(E)** The FC between the left Amyg and the right SFG correlated with headache intensity change rate (*r* = 0.361, *p* < 0.05). MwoA, migraine without aura; FC, functional connectivity; L, left hemisphere; R, right hemisphere; SFG, superior frontal gyrus; MFG, middle frontal gyrus; SPL, superior parietal lobule; IPL, inferior parietal lobule; INS, insular gyrus; CG, cingulate gyrus; Amyg, amygdala; Hipp, hippocampus; Tha, thalamus.

Compared to the pretreatment evaluations, patients with MwoA had decreased FCs between Amyg and INS, Amyg and SFG, CG and SFG, Hipp and SFG, and Tha subregions (*p*-FDR < 0.05). Also, patients with MwoA had increased FCs between Amyg and MFG, Hipp and MFG, Hipp and INS, IPL and INS, IPL and MFG, IPL and SFG, and Tha subregions than pre-acupuncture (*p*-FDR < 0.05) (Figures 3A,B and Supplementary Table 4). Among the significantly different FCs, 2 FCs out of 46 were determined to have significantly correlated with the rate of improvement in headache intensity (Figure 3C). The significantly correlated FCs were (1) FC between the left Amyg and left MFG (*r* = –0.355, *p* < 0.05) (Figure 3D) and (2) FC between the left Amyg and left SFG (*r* = 0.361, *p* < 0.05) (Figure 3E).

#### The Joint Effects Between Headache-Intensity Responders and Non-responders (Between Pre- and Post-acupuncture)

**FIGURE 4:**
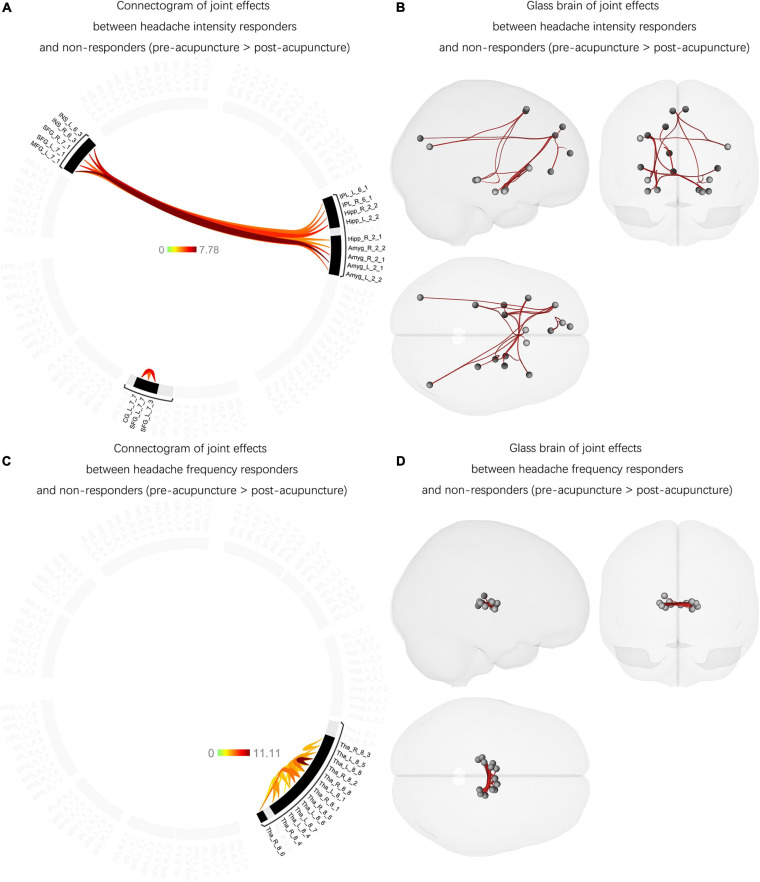
The joint effects between the headache intensity responders, frequency responders, and non-responders (between pre- and post-acupuncture). **(A)** Significantly decreased and increased FCs were found between the headache intensity responders and non-responders compared with post-acupuncture, and the color bar represents the *t*-value of FC. **(B)** Headache intensity responders manifested significantly increased FCs between Amyg and MFG, Amyg and INS, IPL and SFG, IPL and MFG, Hipp and SFG, Hipp and MFG, Hipp and INS, and CG and SFG compared with posttreatment. Red connection lines represent an increased FC before acupuncture than post-acupuncture patients with MwoA. **(C)** Significantly decreased and increased FCs were found between the headache frequency responders and non-responders compared with post-acupuncture, and the color bar represents the *t*-value of FC. **(D)** Headache frequency responders showed significantly increased FC within Tha subregions compared with posttreatment. Red connection lines represent an increased FC before acupuncture than post-acupuncture patients with MwoA. MwoA, migraine without aura; responder, patient with significant clinical improvement; non-responder, patient with nonsignificant clinical improvement; FC, functional connectivity; L, left hemisphere; R, right hemisphere; SFG, superior frontal gyrus; MFG, middle frontal gyrus; SPL, superior parietal lobule; IPL, inferior parietal lobule; INS, insular gyrus; CG, cingulate gyrus; Amyg, amygdala; Hipp, hippocampus; Tha, thalamus.

Joint effects were found between the headache-intensity response groups and non-responders. The *F*-test showed significantly increased FCs between Amyg and MFG, Amyg and INS, IPL and SFG, IPL and MFG, Hipp and SFG, Hipp and MFG, Hipp and INS, and CG and SFG affected acupuncture therapeutic effects compared with posttreatment (*p*-FDR < 0.05) (Figures 4A,B and Supplementary Table 5). Among these FCs, no FC has significantly correlated with headache intensity (*p* > 0.05) and the rate of improvement in headache intensity (*p* > 0.05).

#### The Joint Effects Between the Headache-Frequency Responders and Non-responders (Between Pre- and Post-acupuncture)

Unlike the joint effects between the headache-intensity responders and non-responders, the headache-frequency responders had different unique patterns with increased FCs mainly between the Tha subregions compared with posttreatment (*p*-FDR < 0.05) (Figures 4C,D and Supplementary Table 6). Also, no FC has significantly correlated with headache frequency (*p* > 0.05) and the rate of improvement in headache frequency (*p* > 0.05).

## Discussion

In this study, we investigated the different FC patterns between patients with migraine and HCs. We have also examined the differences before and after acupuncture treatment and the differences between responders and non-responders. As per our findings, it was demonstrated that FCs were altered in participants with migraine compared to HCs, and these altered FCs were mainly in the CG and INS, CG and IPL, CG and SFG, and MFG and IPL. The analysis found that the FC with significant differences mentioned above was restored after acupuncture treatment. Furthermore, significant differences were noted between responders and non-responders. The different FCs between the headache-intensity responder and the non-responder were mainly determined in the Amyg, INS, IPL, SFG, and MFG. In contrast, the different FCs within Tha subregions were found between the headache-frequency responder and the non-responder. Lastly, the correlation analysis suggested a potential correlation between FCs and clinical symptoms.

To date, a large number of neuroimaging studies have demonstrated the structural and functional alterations in patients with migraine. Most of those regions are associated with multiple aspects of pain processing. Several recent resting-state fMRI studies suggested abnormal FC in SN, which was found to be crucial in stimulus detection and allocation of attentional resources (Chong et al., 2016). Schmitz et al. (2008) found that decreased gray matter density in the frontoparietal lobe of patients with migraine negatively correlated with slower responsiveness in a shifting attentional task. It suggested a potential relationship between cognitive function and gray matter deficits in patients with migraine. Mathur et al. (2015) have also investigated the brain activation patterns during cognitive task performance during simultaneous thermal pain stimulation. Interestingly, patients with migraine showed significantly less deactivation in these regions, indicating a potential abnormal regulation of pain-cognitive pathways in patients with migraine. Mickleborough et al. (2016) used an fMRI visual attention orientation task to study brain activation patterns in patients with interictal migraine and HCs. Although patients with migraine performed as well as HCs on the attention task, patients with migraine were found to have decreased activation of the ventral attention network, a region associated with redirecting attention to salient stimuli and belonging to the second-order perceptual matrix of the pain matrix. Compared to separated FC networks, the pain matrix theory emphasizes that the pain experience is caused by the coordinated activity of multiple brain regions, with no single “pain center.” This finding is consistent with the “no single migraine generator” in migraine research. In other words, patients with migraine showed a wide range of intrinsic neuron activity differences, including but not limited to CEN, DMN, and SN alterations.

Moreover, brain activation features can vary between studies, such as increased FC in the periaqueductal gray (PAG), anterior insula, and anterior cingulate cortex with other regions (Tso and Goadsby, 2015). Because how an individual perceives pain is a dynamic process that continuously reconstructs sensations by combining sensory input with memory and internal representations (Garcia-Larrea and Peyron, 2013), separating the different levels of processing helps understand the mechanism. Unlike visual or other systems, the perception of pain activates multiple cortical areas simultaneously, and each of these areas provides a different aspect to the pain experience. Only when all information is integrated into the frontal cortex, pain and behaviors associated with it then begin to emerge. Thus, each region of the pain matrix provides different but essential aspects of the pain experience, starting with nociception, followed by emotional and cognitive–behavioral aspects (Iannetti and Mouraux, 2010).

In this study, FC analysis showed that patients with migraine had decreased FCs in the left MFG and IPL, IPL and SFG, IPL and MFG, IPL and CG, INS, and CG than those in HCs. Conversely, compared to HCs, patients with migraine were observed to have increased FCs in the CG and SFG, IPL, and INS. The most frequent regions between these increased or decreased FCs were associated with CG, followed by the PPC regions, participating in spatial and attention processing. Again, the PFC and insula relate to higher levels of neural activity, and these related regions construct the basic framework for pain stimulation discrimination.

Classical pain models emphasize the role of primary and secondary somatosensory cortices in the processing of sensory features of noxious stimuli but rarely distinguish between different sensory dimensions. Oshiro et al. (2009) indicated that spatial features of stimuli are processed *via* dorsal streams involving PPC and dorsolateral prefrontal cortex (dlPFC). The discrimination of pain intensity is described as a non-spatial sensory feature that activates a network of areas, including the anterior insular cortex and PFC. The model by Oshiro et al. (2009) describes an internal neural activity model under the second-order perceptual matrix and refines the specific functions between different regions, which could complement the pain matrix theory. Similarly, the abnormal neural activity found in this study indicates that the pathological information processing flow exists in patients with migraine. It means a manifestation of the imbalance in neural homeostasis that directly leads to the persistence of incomplete processing of pain information.

In the correlation analysis, the results illustrated that there were potential correlations between clinical symptoms and neurological features. From the perspective of neural activity, the frequency of headache attacks will reduce if the synchronization between CG and SFG is elevated, suggesting there are pathological information processing flows in migraine patients, which directly lead to the persistence of incomplete pain information processing. And this incomplete processing flow may have led to potential pain amplification, known as central sensitization, and manifesting as allodynia in patients with migraine (Strassman et al., 1996). It is widespread in patients with migraine and is considered a marker of migraine chronicity (Scher et al., 2017). In terms of FC, the CG plays an important role in pain management and pain anticipation (Xiao and Zhang, 2018), and activation of CG may contribute to the chronic pain state (Bliss et al., 2016). In addition, CG and SFG were found to exhibit top–down modulation of pain in nociceptive input (Seifert et al., 2013). According to the studies mentioned above, the correlation suggested that the CG and SFG participated in pain processing and perception on the one hand and illustrated that the abnormal FC could be a signature in the chronicity of migraine on the other.

After acupuncture treatment, the abnormal FC of the second-order perceptual matrix in patients with migraine had been restored. Besides, FC analysis indicated that the Hipp, Amyg, and Tha subregions contain increased and decreased FCs, which could be related to the non-specific analgesic effect of acupuncture, indicating that acupuncture treatment affects the limbic system. Studies have suggested that acupuncture has a modulatory effect on the limbic system, which is often associated with the Amyg and Hipp (Fang et al., 2009; Hui et al., 2010; Luo et al., 2020). The Amyg and Hipp form a circuit for sensory and emotional processing (Yang and Wang, 2017) and exhibit structural plasticity associated with headache frequency and prognosis of migraine (Liu et al., 2017).

Acupuncture manipulation begins with stimulation of multiple peripheral sensory receptors activating the somatosensory cortex, which is the same as tactile stimulation of somatosensory overlying areas mediated by activation of the limbic system (Hui et al., 2000). Furthermore, unlike pain modulation processing in the limbic system, emotional processing does not modify pain experience by increasing sensory gain, but rather “reassessing” pain perception (Apkarian et al., 2005). Both the medial PFC and lateral PFC were involved (Mohr et al., 2012). In brief, the networks that acupuncture treatment modulated can modify instant sensations driven by the second-order and third-order matrix. The FCs correlated with clinical symptoms mainly contained in the CG, Amyg, and PFC, which are the regions for integrating pain information and emotional modulation – the final step before pain arises.

Of the 48 participants with migraine, 24 were assigned as headache-intensity responders, whereas 19 were headache-frequency responders based on the acupuncture response definition. In contrast to the differences between pre- and post-treatment FC in all patients, the brain activities differed significantly from the headache-intensity responder. It is mainly characterized by SFG, which is consistent with the pre- and post-treatment differences in all patients but does not contain FC changes within the Tha. PFC activation was associated with pain relief after receiving electroacupuncture. Yang et al. (2012) used fluorodeoxyglucose positron emission tomography to find that acupuncture increased PFC metabolism in patients with acute migraine while reducing pain. Electroacupuncture has been shown to modulate functional activity in dopaminergic regions in a time-dependent way (Napadow et al., 2009; Maeda et al., 2013), suggesting that part of the mechanism of acupuncture analgesia is related to modulating pain sensitivity by prefrontal dopamine levels (Dent and Neill, 2012).

The acupuncture modulation of these regions is the specific neural mechanism of acupuncture for patients with migraine, while the involvement of other mechanisms was also found in this study. Because every acupuncture operation is a stimulation or pain signal (Theysohn et al., 2014) inducing the activation of different brain regions (Kufahl et al., 2008; Chae et al., 2013), they closely rely on “acupoints” and “*deqi*.” Acupuncture has been determined to exhibit significant analgesic effects only when patients experience the sensation of *deqi* (a sensation of soreness, numbness, heaviness, and distension) (Hui et al., 2000). There is a mainstream view that acupuncture signals ascend to the cerebral cortex mainly through the ventral lateral nerve of the spinal cord because many nuclei are involved in acupuncture signal processing and form a complex network, including but not limited to the Tha. This finding means that pathways integrate afferent pain stimulation and acupuncture stimulation in the central nervous system simultaneously. Also, the results showed that the Tha-related FCs partially fit with the neurological features of headache-frequency responder. Tu et al. (2019) verified that abnormalities in the thalamocortical network could disrupt sensory, cognitive, and motor neural processes in patients with migraine. Recent studies have found that thalamic plasticity correlates with migraine chronicity (Chen et al., 2020) and is associated with the frequency of headaches (Hodkinson et al., 2016). The above study is consistent with our results and suggests an important role of the Tha in influencing the frequency of migraine.

In contrast, brain areas associated with the limbic system were found to be more associated with headache attacks in patients with migraine. In the pain processing networks, pain discrimination processes are controlled by the limbic system processing, which is more related to the neuropathological mechanisms at the beginning of the migraine – indicating that pain discrimination and integration processes are the basis of migraine attacks. As the disease progresses, this function’s allostasis is overloaded before the limbic system, followed by central sensitization (Noseda and Burstein, 2013; Russo et al., 2018). Subsequently, the limbic system allostasis overloads under the influence of long-term repetition, and migraine then enters a shift to chronicity.

Central nervous system alterations in pain processing-related functions were also found in patients with chronic pain, thus representing the non-specific effects of the disease. The pathological basis of these changes includes adaptive remodeling of neural pathways and secondary reactive gliosis in brain regions (Lakhan et al., 2013). FC patterns of migraine change over time in individuals, but these can be assessed by repeated imaging at multiple time points. Liu et al. (2013) studied structural changes in adult patients with migraine over 1 year, with patients exhibiting a loss of gray matter structural volume. Another 3-year follow-up study by Erdelyi-Botor et al. (2015) showed progression of high white matter intensities in patients with migraine. These changes were determined to be more common in patients with high-frequency migraine than with low-frequency migraine (Erdelyi-Botor et al., 2015). These studies reveal structural and functional changes in the brains of patients with migraine over time and that headache attacks are closely related to the progression of brain changes.

Unlike previous studies (Li et al., 2017a,b), this retrospective study intends to investigate the potential modulatory effects of acupuncture in pathological states. This study hypothesized an abnormal pathological pattern in patients with migraine and selected pain processing-related regions as ROIs. Also, for the first time, this study divided the subgroup analysis into two dimensions: improvement in headache intensity and improvement in headache frequency. However, the weak correlation found in this study may be due to insufficient sample size or related to the design of ROIs. In other words, the ROIs used in this study do not evoke pain under direct stimulation but rather produce nociception through the combination of multiple pieces of information (Garcia-Larrea and Peyron, 2013). The correlation between this indirect and complex pain processing progress and clinical symptoms needs to be further explored.

In conclusion, this study has demonstrated that abnormal central neural activity patterns of the pain processing-related regions exist in patients with migraine, and this abnormal activity is primarily in the frontal, parietal, and limbic regions, suggesting that impaired integration of pain spatial and intensity information could be a typical central pathological feature of patients with MwoA. Thus, acupuncture can relieve headache symptoms by regulating the brain regions above as a specific therapeutic effect and modulating neural activity in brain regions related to the nociceptive areas as non-specific analgesics.

## Limitations

Determining the specificity of acupuncture treatment in pain mechanism is inadequate for a single study; thus, future studies should focus on comparing other headache types and other pain types to clarify the specificity of acupuncture. In addition, a possible association between duration and frequency improvement of headache was found during this study, suggesting a relationship between migraine duration and central mechanisms, which could be a topic of future research.

## Data Availability Statement

The raw data supporting the conclusions of this article will be made available by the authors, without undue reservation.

## Ethics Statement

The studies involving human participants were reviewed and approved by the Ethics Committee of the First Teaching Hospital of Chengdu University of Traditional Chinese Medicine. The patients/participants provided their written informed consent to participate in this study.

## Author Contributions

LL and FZ conceived and designed the study. QX, GH, JC, and SW recruited the participants. ZT and TY analyzed the data. ZT drafted the manuscript. YG, LL, and FZ revised the manuscript. All authors contributed to the article and approved the submitted version.

## Conflict of Interest

The authors declare that the research was conducted in the absence of any commercial or financial relationships that could be construed as a potential conflict of interest.

## Publisher’s Note

All claims expressed in this article are solely those of the authors and do not necessarily represent those of their affiliated organizations, or those of the publisher, the editors and the reviewers. Any product that may be evaluated in this article, or claim that may be made by its manufacturer, is not guaranteed or endorsed by the publisher.
