# Supplementary material for: Acupuncture Modulation Effect on Pain Processing Patterns in Patients With Migraine Without Aura
Source: Front Neurosci. 2021 Aug 26;15:729218. doi: 10.3389/fnins.2021.729218 (PMC8427167; doi:10.3389/fnins.2021.729218)
Supplement: Supplementary file 1 [file Data_Sheet_1.docx]

Supplementary Material

# Supplementary 1 | ROI Design

| Lobe | Gyrus | Left and Right Hemisphere | Label ID.L | Label ID.R | Anatomical and modified Cyto-architectonic descriptions | lh.MNI(X,Y,Z) | rh.MNI(X,Y,Z) |
| --- | --- | --- | --- | --- | --- | --- | --- |
| Frontal Lobe | SFG, Superior Frontal Gyrus | SFG_L(R)_7_1 | 1 | 2 | A8m, medial area 8 | -5 ,15, 54 | 7, 16, 54 |
|  |  | SFG_L(R)_7_2 | 3 | 4 | A8dl, dorsolateral area 8 | -18, 24, 53 | 22, 26, 51 |
|  |  | SFG_L(R)_7_3 | 5 | 6 | A9l, lateral area 9 | -11, 49, 40 | 13, 48, 40 |
|  |  | SFG_L(R)_7_4 | 7 | 8 | A6dl, dorsolateral area 6 | -18, -1, 65 | 20, 4, 64 |
|  |  | SFG_L(R)_7_5 | 9 | 10 | A6m, medial area 6 | -6, -5, 58 | 7, -4, 60 |
|  |  | SFG_L(R)_7_6 | 11 | 12 | A9m,medial area 9 | -5, 36, 38 | 6, 38, 35 |
|  |  | SFG_L(R)_7_7 | 13 | 14 | A10m, medial area 10 | -8, 56, 15 | 8, 58, 13 |
|  | MFG, Middle Frontal Gyrus | MFG_L(R)_7_1 | 15 | 16 | A9/46d, dorsal area 9/46 | -27, 43, 31 | 30, 37, 36 |
|  |  | MFG_L(R)_7_2 | 17 | 18 | IFJ, inferior frontal junction | -42, 13, 36 | 42, 11, 39 |
|  |  | MFG_L(R)_7_3 | 19 | 20 | A46, area 46 | -28, 56, 12 | 28, 55, 17 |
|  |  | MFG_L(R)_7_4 | 21 | 22 | A9/46v, ventral area 9/46 | -41, 41, 16 | 42, 44, 14 |
|  |  | MFG_L(R)_7_5 | 23 | 24 | A8vl, ventrolateral area 8 | -33, 23, 45 | 42, 27, 39 |
|  |  | MFG_L(R)_7_6 | 25 | 26 | A6vl, ventrolateral area 6 | -32, 4, 55 | 34, 8, 54 |
|  |  | MFG_L(R)_7_7 | 27 | 28 | A10l, lateral area10 | -26, 60, -6 | 25, 61, -4 |
| Parietal Lobe | SPL, Superior Parietal Lobule | SPL_L(R)_5_1 | 125 | 126 | A7r, rostral area 7 | -16, -60, 63 | 19, -57, 65 |
|  |  | SPL_L(R)_5_2 | 127 | 128 | A7c, caudal area 7 | -15, -71, 52 | 19, -69, 54 |
|  |  | SPL_L(R)_5_3 | 129 | 130 | A5l, lateral area 5 | -33, -47, 50 | 35, -42, 54 |
|  |  | SPL_L(R)_5_4 | 131 | 132 | A7pc, postcentral area 7 | -22, -47, 65 | 23, -43, 67 |
|  |  | SPL_L(R)_5_5 | 133 | 134 | A7ip, intraparietal area 7(hIP3) | -27, -59, 54 | 31, -54, 53 |
|  | IPL, Inferior Parietal Lobule | IPL_L(R)_6_1 | 135 | 136 | A39c, caudal area 39(PGp) | -34, -80, 29 | 45, -71, 20 |
|  |  | IPL_L(R)_6_2 | 137 | 138 | A39rd, rostrodorsal area 39(Hip3) | -38, -61, 46 | 39, -65, 44 |
|  |  | IPL_L(R)_6_3 | 139 | 140 | A40rd, rostrodorsal area 40(PFt) | -51, -33, 42 | 47, -35, 45 |
|  |  | IPL_L(R)_6_4 | 141 | 142 | A40c, caudal area 40(PFm) | -56, -49, 38 | 57, -44, 38 |
|  |  | IPL_L(R)_6_5 | 143 | 144 | A39rv, rostroventral area 39(PGa) | -47, -65, 26 | 53, -54, 25 |
|  |  | IPL_L(R)_6_6 | 145 | 146 | A40rv, rostroventral area 40(PFop) | -53, -31, 23 | 55, -26, 26 |
| Insular Lobe | INS, Insular Gyrus | INS_L(R)_6_1 | 163 | 164 | G, hypergranular insula | -36, -20, 10 | 37, -18, 8 |
|  |  | INS_L(R)_6_2 | 165 | 166 | vIa, ventral agranular insula | -32, 14, -13 | 33, 14, -13 |
|  |  | INS_L(R)_6_3 | 167 | 168 | dIa, dorsal agranular insula | -34, 18, 1 | 36, 18, 1 |
|  |  | INS_L(R)_6_4 | 169 | 170 | vId/vIg, ventral dysgranular and granular insula | -38, -4, -9 | 39, -2, -9 |
|  |  | INS_L(R)_6_5 | 171 | 172 | dIg, dorsal granular insula | -38, -8, 8 | 39, -7, 8 |
|  |  | INS_L(R)_6_6 | 173 | 174 | dId, dorsal dysgranular insula | -38, 5, 5 | 38, 5, 5 |
| Limbic Lobe | CG, Cingulate Gyrus | CG_L(R)_7_1 | 175 | 176 | A23d, dorsal area 23 | -4, -39, 31 | 4, -37, 32 |
|  |  | CG_L(R)_7_2 | 177 | 178 | A24rv, rostroventral area 24 | -3, 8, 25 | 5, 22, 12 |
|  |  | CG_L(R)_7_3 | 179 | 180 | A32p, pregenual area 32 | -6, 34, 21 | 5, 28, 27 |
|  |  | CG_L(R)_7_4 | 181 | 182 | A23v, ventral area 23 | -8, -47, 10 | 9, -44, 11 |
|  |  | CG_L(R)_7_5 | 183 | 184 | A24cd, caudodorsal area 24 | -5, 7, 37 | 4, 6, 38 |
|  |  | CG_L(R)_7_6 | 185 | 186 | A23c, caudal area 23 | -7, -23, 41 | 6, -20, 40 |
|  |  | CG_L(R)_7_7 | 187 | 188 | A32sg, subgenual area 32 | -4, 39, -2 | 5, 41, 6 |
| Subcortical Nuclei | Amyg, Amygdala | Amyg_L(R)_2_1 | 211 | 212 | mAmyg, medial amygdala | -19, -2, -20 | 19, -2, -19 |
|  |  | Amyg_L(R)_2_2 | 213 | 214 | lAmyg, lateral amygdala | -27, -4, -20 | 28, -3, -20 |
|  | Hipp, Hippocampus | Hipp_L(R)_2_1 | 215 | 216 | rHipp, rostral hippocampus | -22, -14, -19 | 22, -12, -20 |
|  |  | Hipp_L(R)_2_2 | 217 | 218 | cHipp, caudal hippocampus | -28, -30, -10 | 29, -27, -10 |
|  | Tha, Thalamus | Tha_L(R)_8_1 | 231 | 232 | mPFtha, medial pre-frontal thalamus | -7, -12, 5 | 7, -11, 6 |
|  |  | Tha_L(R)_8_2 | 233 | 234 | mPMtha, pre-motor thalamus | -18, -13, 3 | 12, -14, 1 |
|  |  | Tha_L(R)_8_3 | 235 | 236 | Stha, sensory thalamus | -18, -23, 4 | 18, -22, 3 |
|  |  | Tha_L(R)_8_4 | 237 | 238 | rTtha, rostral temporal thalamus | -7, -14, 7 | 3, -13, 5 |
|  |  | Tha_L(R)_8_5 | 239 | 240 | PPtha, posterior parietal thalamus | -16, -24, 6 | 15, -25, 6 |
|  |  | Tha_L(R)_8_6 | 241 | 242 | Otha, occipital thalamus | -15, -28, 4 | 13, -27, 8 |
|  |  | Tha_L(R)_8_7 | 243 | 244 | cTtha, caudal temporal thalamus | -12, -22, 13 | 10, -14, 14 |
|  |  | Tha_L(R)_8_8 | 245 | 246 | lPFtha, lateral pre-frontal thalamus | -11, -14, 2 | 13, -16, 7 |

L, left hemisphere; R, right hemisphere; SFG, superior frontal gyrus; MFG, middle frontal gyrus; SPL, superior parietal lobule; IPL, inferior parietal lobule; INS, insular gyrus; CG, cingulate gyrus; Amyg, amygdala; Hipp, hippocampus; Tha, thalamus.

# Supplementary 2 | Quality Assurance Plot


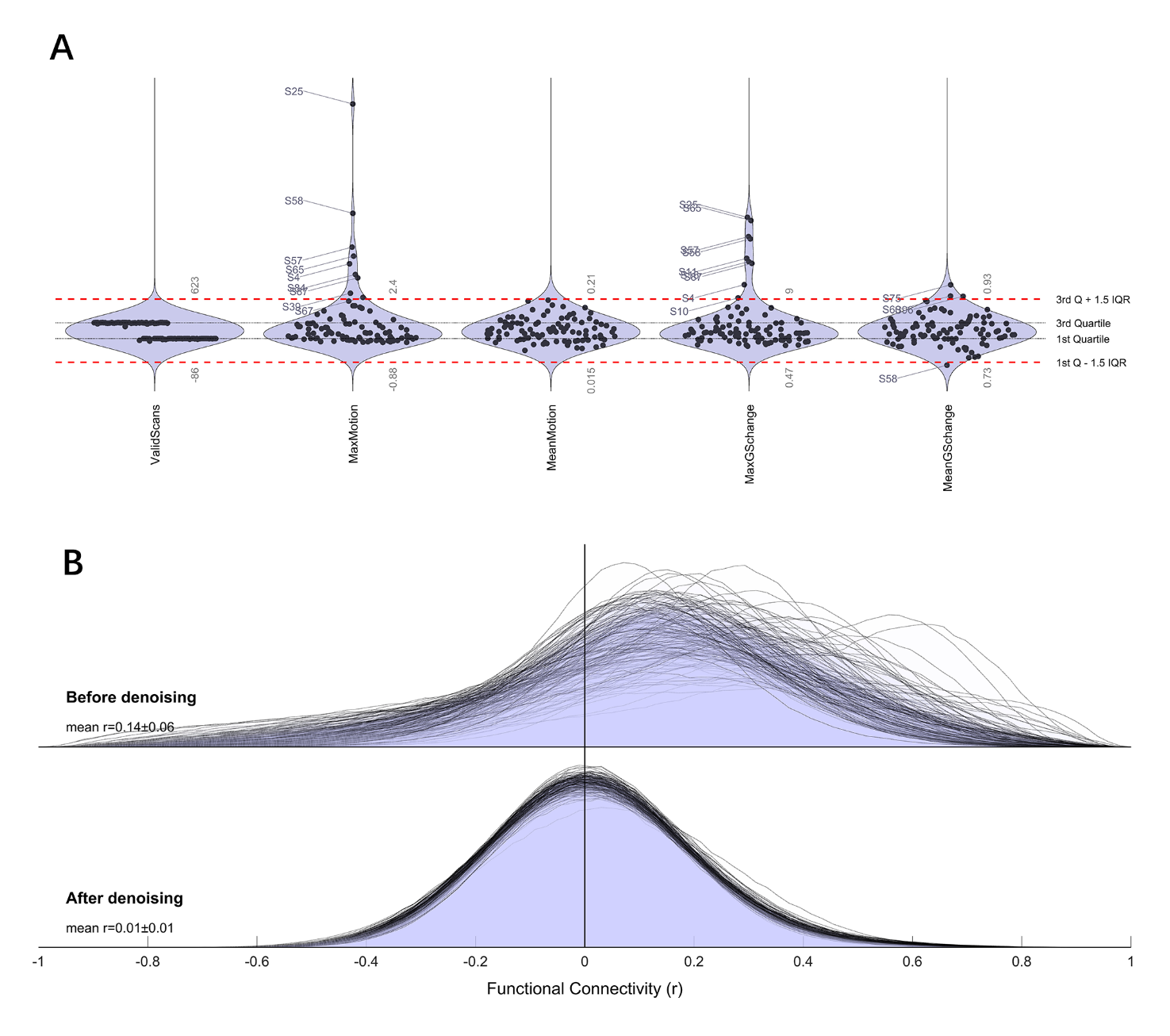


(**A**) Quality assurance was checked before functional connectivity analyses, and no MwoA patients or HCs were excluded depend on the number of volumes in which head position was 0.5 mm different from adjacent volumes was more than 20%. (**B**) After the denoising process, the stability of the functional connection was improved.

# Supplementary 3 | The Neuro Activities Difference Between Migraineurs and HCs

| Analysis | Statistic | p-unc | p-FDR |
| --- | --- | --- | --- |
| Cluster 1/120 | F(3,92) = 7.11 | 0.000239 | 0.026737 |
| Connection CG_L_7_4-IPL_R_6_6 | T(94) = 3.97 | 0.00014 | 0.004609 |
| Connection CG_L_7_4-INS_R_6_1 | T(94) = 3.21 | 0.001789 | 0.029514 |
| Connection CG_L_7_4-IPL_L_6_6 | T(94) = 2.86 | 0.005228 | 0.064702 |
| Connection CG_R_7_4-INS_R_6_1 | T(94) = 2.99 | 0.003551 | 0.070309 |
| Connection CG_R_7_4-IPL_R_6_6 | T(94) = 2.91 | 0.004449 | 0.073413 |
| Connection CG_L_7_4-INS_R_6_4 | T(94) = 2.53 | 0.013053 | 0.129228 |
| Connection CG_L_7_4-INS_L_6_1 | T(94) = 2.18 | 0.032098 | 0.186923 |
| Connection CG_R_7_4-IPL_L_6_6 | T(94) = 2.12 | 0.036706 | 0.363393 |
| Connection IPL_L_6_5-INS_L_6_4 | T(94) = -2.07 | 0.041327 | 0.381351 |
| Cluster 2/120 | F(3,92) = 6.58 | 0.000446 | 0.026737 |
| Connection MFG_L_7_5-IPL_L_6_5 | T(94) = 2.77 | 0.006793 | 0.096074 |
| Connection IPL_L_6_2-IPL_L_6_5 | T(94) = 3.18 | 0.001985 | 0.19655 |
| Connection MFG_L_7_2-IPL_L_6_5 | T(94) = 2.23 | 0.028192 | 0.348874 |
| Connection MFG_L_7_7-IPL_L_6_5 | T(94) = 2.45 | 0.016076 | 0.5305 |
| Cluster 3/120 | F(3,92) = 5.70 | 0.001271 | 0.046712 |
| Connection MFG_L_7_5-MFG_L_7_2 | T(94) = 4.93 | 0.000004 | 0.000352 |
| Connection MFG_L_7_5-IPL_L_6_2 | T(94) = 2.21 | 0.029444 | 0.264997 |
| Connection MFG_L_7_7-MFG_L_7_2 | T(94) = 2.72 | 0.007698 | 0.5305 |
| Cluster 4/120 | F(3,92) = 5.35 | 0.001922 | 0.046712 |
| Connection CG_L_7_4-SFG_L_7_1 | T(94) = -4.89 | 0.000004 | 0.000411 |
| Connection CG_R_7_4-SFG_L_7_1 | T(94) = -4.73 | 0.000008 | 0.000779 |
| Connection CG_L_7_4-SFG_R_7_1 | T(94) = -3.71 | 0.00035 | 0.008659 |
| Connection CG_R_7_4-SFG_R_7_1 | T(94) = -3.55 | 0.000612 | 0.030292 |
| Connection CG_R_7_1-SFG_L_7_1 | T(94) = -2.91 | 0.004563 | 0.22588 |
| Connection CG_R_7_1-SFG_R_7_1 | T(94) = -2.04 | 0.044539 | 0.440932 |
| Connection CG_L_7_1-SFG_L_7_1 | T(94) = -2.51 | 0.013927 | 0.459594 |
| Cluster 5/120 | F(2,93) = 6.68 | 0.001946 | 0.046712 |
| Connection SFG_R_7_2-IPL_R_6_5 | T(94) = 3.28 | 0.001441 | 0.142614 |
| Connection IPL_R_6_2-IPL_R_6_5 | T(94) = 2.61 | 0.010443 | 0.258466 |

L, left hemisphere; R, right hemisphere; HC, healthy control; SFG, superior frontal gyrus; MFG, middle frontal gyrus; SPL, superior parietal lobule; IPL, inferior parietal lobule; INS, insular gyrus; CG, cingulate gyrus.

# Supplementary 4 | The Neuro Activities Difference Before and After Acupuncture Treatment

| Analysis Unit | Statistic | p-unc | p-FDR |
| --- | --- | --- | --- |
| Cluster 1/91 | F(2,46) = 9.99 | 0.00025 | 0.022626 |
| Connection Hipp_L_2_2-SFG_L_7_1 | T(47) = 3.27 | 0.00202 | 0.056255 |
| Connection Amyg_L_2_1-INS_R_6_3 | T(47) = 3.56 | 0.000856 | 0.084793 |
| Connection IPL_R_6_1-MFG_L_7_1 | T(47) = -2.94 | 0.005143 | 0.084898 |
| Connection IPL_R_6_1-SFG_R_7_1 | T(47) = -2.94 | 0.005145 | 0.084898 |
| Connection Hipp_L_2_2-INS_L_6_3 | T(47) = -2.53 | 0.014765 | 0.094761 |
| Connection Amyg_L_2_1-MFG_L_7_1 | T(47) = -3.16 | 0.002742 | 0.11919 |
| Connection Hipp_R_2_2-SFG_L_7_1 | T(47) = 2.70 | 0.0096 | 0.211731 |
| Connection Amyg_L_2_1-INS_L_6_3 | T(47) = 2.17 | 0.035038 | 0.315343 |
| Connection Amyg_R_2_2-INS_R_6_3 | T(47) = 2.11 | 0.040651 | 0.328501 |
| Connection Amyg_L_2_1-SFG_R_7_1 | T(47) = 2.09 | 0.042155 | 0.347783 |
| Connection Hipp_R_2_1-MFG_L_7_1 | T(47) = -2.76 | 0.008276 | 0.356896 |
| Connection IPL_L_6_1-MFG_L_7_1 | T(47) = -2.86 | 0.006276 | 0.547549 |
| Connection IPL_L_6_1-INS_L_6_3 | T(47) = -2.19 | 0.033276 | 0.547549 |
| Connection Amyg_L_2_2-MFG_L_7_1 | T(47) = -2.25 | 0.028881 | 0.553057 |
| Connection Amyg_L_2_2-SFG_R_7_1 | T(47) = 2.18 | 0.034204 | 0.553057 |
| Connection Amyg_R_2_1-INS_R_6_3 | T(47) = 2.01 | 0.049867 | 0.627566 |
| Connection Hipp_L_2_1-MFG_L_7_1 | T(47) = -2.22 | 0.031383 | 0.723659 |
| Cluster 2/91 | F(2,46) = 9.02 | 0.000497 | 0.022626 |
| Connection Tha_R_8_8-Tha_R_8_2 | T(47) = -4.69 | 0.000024 | 0.002338 |
| Connection Tha_L_8_1-Tha_R_8_2 | T(47) = -3.40 | 0.001375 | 0.105309 |
| Connection Tha_L_8_1-Tha_L_8_8 | T(47) = -3.13 | 0.003037 | 0.105309 |
| Connection Tha_R_8_2-Tha_L_8_8 | T(47) = -2.63 | 0.011646 | 0.158726 |
| Connection Tha_R_8_2-Tha_L_8_5 | T(47) = -2.44 | 0.018316 | 0.161062 |
| Connection Tha_R_8_8-Tha_L_8_8 | T(47) = -3.02 | 0.004061 | 0.201031 |
| Connection Tha_R_8_1-Tha_R_8_2 | T(47) = -2.99 | 0.004472 | 0.216986 |
| Connection Tha_R_8_1-Tha_R_8_8 | T(47) = -2.67 | 0.01038 | 0.216986 |
| Connection Tha_L_8_1-Tha_R_8_8 | T(47) = -2.58 | 0.012914 | 0.249663 |
| Connection Tha_L_8_3-Tha_L_8_2 | T(47) = -2.29 | 0.026694 | 0.277974 |
| Connection Tha_R_8_4-Tha_L_8_4 | T(47) = -3.14 | 0.002954 | 0.292409 |
| Connection Tha_R_8_5-Tha_R_8_2 | T(47) = -3.09 | 0.003327 | 0.329396 |
| Connection Tha_R_8_4-Tha_L_8_1 | T(47) = -2.68 | 0.010146 | 0.333636 |
| Connection Tha_L_8_6-Tha_R_8_2 | T(47) = -2.98 | 0.004501 | 0.445635 |
| Connection Tha_L_8_6-Tha_R_8_8 | T(47) = -2.72 | 0.009203 | 0.455546 |
| Connection Tha_L_8_6-Tha_L_8_1 | T(47) = -2.52 | 0.015131 | 0.475911 |
| Connection Tha_L_8_6-Tha_L_8_5 | T(47) = -2.42 | 0.019229 | 0.475911 |
| Connection Tha_L_8_5-Tha_R_8_3 | T(47) = -2.79 | 0.007679 | 0.50962 |
| Connection Tha_L_8_5-Tha_L_8_3 | T(47) = -2.23 | 0.030886 | 0.50962 |
| Connection Tha_R_8_1-Tha_L_8_8 | T(47) = -2.03 | 0.047642 | 0.589572 |
| Connection Tha_R_8_7-Tha_L_8_2 | T(47) = 2.32 | 0.024714 | 0.661137 |
| Connection Tha_R_8_5-Tha_R_8_1 | T(47) = -2.55 | 0.014055 | 0.695734 |
| Connection Tha_L_8_6-Tha_R_8_3 | T(47) = -2.12 | 0.039115 | 0.774481 |
| Connection Tha_R_8_6-Tha_R_8_1 | T(47) = -2.71 | 0.009267 | 0.917482 |
| Connection Tha_L_8_7-Tha_L_8_5 | T(47) = -2.07 | 0.044377 | 0.952943 |
| Cluster 3/91 | F(2,46) = 8.15 | 0.000935 | 0.028365 |
| Connection CG_L_7_7-SFG_L_7_3 | T(47) = 3.46 | 0.001156 | 0.02289 |
| Connection CG_L_7_7-SFG_L_7_7 | T(47) = 2.90 | 0.005726 | 0.056278 |
| Connection CG_L_7_7-SFG_R_7_7 | T(47) = 2.29 | 0.026714 | 0.126419 |
| Connection CG_L_7_7-SFG_R_7_3 | T(47) = 2.19 | 0.033735 | 0.145207 |

L, left hemisphere; R, right hemisphere; SFG, superior frontal gyrus; MFG, middle frontal gyrus; SPL, superior parietal lobule; IPL, inferior parietal lobule; INS, insular gyrus; CG, cingulate gyrus; Amyg, amygdala; Hipp, hippocampus; Tha, thalamus.

# Supplementary 5 | The Joint Effects Between the Headache Intensity Responders and Nonresponders (Between Pre-And Post-Acupuncture)

| Analysis Unit | Statistic | p-unc | p-FDR |
| --- | --- | --- | --- |
| Cluster 1/91 | F(4,90) = 5.96 | 0.000266 | 0.02418 |
| Connection Amyg_L_2_1-MFG_L_7_1 | F(2,46) = 7.78 | 0.001232 | 0.072206 |
| Connection Amyg_L_2_1-INS_L_6_3 | F(2,46) = 6.55 | 0.003147 | 0.072206 |
| Connection Amyg_L_2_1-INS_R_6_3 | F(2,46) = 6.49 | 0.003281 | 0.072206 |
| Connection IPL_R_6_1-SFG_R_7_1 | F(2,46) = 4.26 | 0.020038 | 0.156685 |
| Connection IPL_R_6_1-MFG_L_7_1 | F(2,46) = 4.23 | 0.020607 | 0.156685 |
| Connection Hipp_L_2_2-SFG_L_7_1 | F(2,46) = 5.23 | 0.00897 | 0.209554 |
| Connection Hipp_L_2_2-INS_L_6_3 | F(2,46) = 3.56 | 0.036626 | 0.281469 |
| Connection Hipp_R_2_2-SFG_L_7_1 | F(2,46) = 4.43 | 0.017334 | 0.281532 |
| Connection Amyg_R_2_1-INS_L_6_3 | F(2,46) = 4.91 | 0.011648 | 0.314929 |
| Connection Amyg_R_2_2-INS_L_6_3 | F(2,46) = 3.57 | 0.036268 | 0.374523 |
| Connection Amyg_R_2_1-INS_R_6_3 | F(2,46) = 3.30 | 0.045895 | 0.40841 |
| Connection Hipp_R_2_1-MFG_L_7_1 | F(2,46) = 3.78 | 0.03009 | 0.411536 |
| Connection Amyg_L_2_2-MFG_L_7_1 | F(2,46) = 4.39 | 0.018014 | 0.594467 |
| Connection IPL_L_6_1-MFG_L_7_1 | F(2,46) = 4.03 | 0.024343 | 0.663356 |
| Cluster 2/91 | F(4,90) = 5.08 | 0.000979 | 0.044561 |
| Connection CG_L_7_7-SFG_L_7_3 | F(2,46) = 6.00 | 0.004837 | 0.095767 |
| Connection CG_L_7_7-SFG_L_7_7 | F(2,46) = 4.12 | 0.022571 | 0.186214 |

L, left hemisphere; R, right hemisphere; SFG, superior frontal gyrus; MFG, middle frontal gyrus; SPL, superior parietal lobule; IPL, inferior parietal lobule; INS, insular gyrus; CG, cingulate gyrus; Amyg, amygdala; Hipp, hippocampus.

# Supplementary 6 | The Joint Effects Between the Headache Frequency Responders and Nonresponders (Between Pre-And Post-Acupuncture)

| Analysis Unit | Statistic | p-unc | p-FDR |
| --- | --- | --- | --- |
| Cluster 1/91 | F(4,90) = 5.90 | 0.000292 | 0.026547 |
| Connection Tha_R_8_8-Tha_R_8_2 | F(2,46) = 11.11 | 0.000116 | 0.011491 |
| Connection Tha_R_8_1-Tha_R_8_2 | F(2,46) = 4.60 | 0.015095 | 0.181277 |
| Connection Tha_R_8_1-Tha_R_8_8 | F(2,46) = 3.76 | 0.030739 | 0.23409 |
| Connection Tha_R_8_2-Tha_L_8_8 | F(2,46) = 4.13 | 0.022493 | 0.318118 |
| Connection Tha_L_8_5-Tha_R_8_3 | F(2,46) = 4.37 | 0.018353 | 0.344463 |
| Connection Tha_R_8_8-Tha_L_8_8 | F(2,46) = 4.53 | 0.016047 | 0.361054 |
| Connection Tha_R_8_5-Tha_R_8_1 | F(2,46) = 5.78 | 0.005783 | 0.392275 |
| Connection Tha_R_8_5-Tha_R_8_2 | F(2,46) = 4.89 | 0.011887 | 0.392275 |
| Connection Tha_L_8_1-Tha_R_8_2 | F(2,46) = 5.79 | 0.005712 | 0.446646 |
| Connection Tha_L_8_1-Tha_L_8_8 | F(2,46) = 4.85 | 0.012222 | 0.446646 |
| Connection Tha_L_8_1-Tha_R_8_8 | F(2,46) = 3.27 | 0.04685 | 0.459543 |
| Connection Tha_R_8_6-Tha_R_8_1 | F(2,46) = 5.94 | 0.005062 | 0.501177 |
| Connection Tha_L_8_6-Tha_L_8_5 | F(2,46) = 4.57 | 0.015537 | 0.568705 |
| Connection Tha_L_8_6-Tha_R_8_2 | F(2,46) = 4.36 | 0.01845 | 0.568705 |
| Connection Tha_L_8_6-Tha_R_8_8 | F(2,46) = 4.10 | 0.022954 | 0.568705 |
| Connection Tha_R_8_4-Tha_L_8_4 | F(2,46) = 5.06 | 0.010344 | 0.572924 |
| Connection Tha_R_8_4-Tha_L_8_1 | F(2,46) = 3.52 | 0.037871 | 0.572924 |
| Connection Tha_R_8_6-Tha_L_8_1 | F(2,46) = 4.16 | 0.021774 | 0.611413 |
| Connection Tha_R_8_6-Tha_L_8_6 | F(2,46) = 4.10 | 0.022978 | 0.611413 |
| Connection Tha_R_8_5-Tha_L_8_1 | F(2,46) = 3.84 | 0.028577 | 0.707285 |
| Connection BTha_R_8_6-Tha_L_8_4 | F(2,46) = 3.39 | 0.04227 | 0.836941 |
| Connection Tha_L_8_7-Tha_L_8_5 | F(2,46) = 3.22 | 0.049099 | 0.892895 |

L, left hemisphere; R, right hemisphere; Tha, thalamus.
